# Short‐Term Multicomponent Exercise Impact on Muscle Function and Structure in Hospitalized Older at Risk of Acute Sarcopenia

**DOI:** 10.1002/jcsm.13602

**Published:** 2024-10-13

**Authors:** Mikel L. Sáez de Asteasu, Nicolás Martínez‐Velilla, Fabricio Zambom‐Ferraresi, Yesenia García‐Alonso, Arkaitz Galbete, Robinson Ramírez‐Vélez, Eduardo L. Cadore, Mikel Izquierdo

**Affiliations:** ^1^ Navarrabiomed Hospital Universitario de Navarra (HUN)‐Universidad Pública de Navarra (UPNA), IdiSNA Pamplona Spain; ^2^ CIBER of Frailty and Healthy Aging (CIBERFES) Instituto de Salud Carlos III Madrid Spain; ^3^ Department of Geriatric Hospital Universitario de Navarra (HUN) Pamplona Spain; ^4^ Exercise Research Laboratory, School of Physical Education, Physiotherapy and Dance Universidade Federal Do Rio Grande Do Sul Porto Alegre Brazil

**Keywords:** Hospitalization, Multicomponent training, Vivifrail

## Abstract

**Background:**

Hospitalization exacerbates sarcopenia and physical dysfunction in older adults. Whether tailored inpatient exercise prevents acute sarcopenia is unknown. This study aimed to examine the effect of a multicomponent exercise programme on muscle and physical function in hospitalized older adults. We hypothesized that participation in a brief tailored exercise regimen (i.e., 3–5 days) would attenuate muscle function and structure changes compared with usual hospital care alone.

**Methods:**

This randomized clinical trial with blinded outcome assessment was conducted from May 2018 to April 2021 at Hospital Universitario de Navarra, Spain. Participants were 130 patients aged 75 years and older admitted to an acute care geriatric unit. Patients were randomized to a tailored 3‐ to 5‐day exercise programme (*n* = 64) or usual hospital care (control, *n* = 66) consisting of physical therapy if needed. The coprimary endpoints were between‐group differences in changes in short physical performance battery (SPPB) score and usual gait velocity from hospital admission to discharge. Secondary endpoints included changes in rectus femoris echo intensity, cross‐sectional area, thickness and subcutaneous and intramuscular fat by ultrasound.

**Results:**

Among 130 randomized patients (mean [SD] age, 87.7 [4.6] years; 57 [44%] women), the exercise group increased their mean SPPB score by 0.98 points (95% CI, 0.28–1.69 points) and gait velocity by 0.09 m/s (95% CI, 0.03–0.15 m/s) more than controls (both *p* < 0.01). No between‐group differences were observed in any ultrasound muscle outcomes. There were no study‐related adverse events.

**Conclusions:**

Three to 5 days of tailored multicomponent exercise provided functional benefits but did not alter muscle or fat architecture compared with usual hospital care alone among vulnerable older patients. Brief exercise may help prevent acute sarcopenia during hospitalization.

**Trial Registration:**

ClinicalTrials.gov identifier: NCT04600453

## 1 Introduction

Consistent evidence indicates that prolonged bed rest while hospitalized accelerates physical dysfunction and sarcopenia in older adults. Fifty per cent of individuals aged 65 years and older develop new disabilities in activities of daily living after hospitalization, termed hospital‐acquired disability (HAD) [1]. The HAD syndrome is independently associated with adverse consequences, including loss of independence, greater health care burden, caregiver stress, institutionalization and mortality [2].

Low physical activity and mobility during hospitalization exacerbate losses in muscle mass and function, which directly worsen physical performance [3]. Typical consequences of prolonged bed rest include dramatic reductions in muscle cross‐sectional area, strength and power [4]. Lower muscle mass is associated with higher post‐discharge mortality in older adults [5]. Hospitalization appears to precipitate acute sarcopenia, defined as accelerated inflammation‐induced changes in muscle mass and strength [6]. Acute sarcopenia is linked with longer hospital stays [7]. Multiple studies show marked muscle strength and muscle mass declines during hospitalization [8, 9, 10], a core domains of consensus sarcopenia criteria [11].

Evidence increasingly supports the essential role of physical activity and exercise during hospitalization for preventing new disability [12, 13, 14]. Multicomponent programmes emphasizing progressive resistance training using machines and/or weights and gait/balance training appear most effective for preserving physical function (e.g., gait speed and SPPB) and muscle strength in hospitalized older patients compared with simple mobility interventions [13, 14], whereas simple mobility exercises are not effective [15]. However, no studies have specifically tested whether structured exercise prevents measurable sarcopenia or improves muscle size and quality in patients during short‐periods of hospitalization in acute geriatric units. A Phase 1 clinical trial reported that a multi‐arm intervention, administered post‐discharge, aimed at increasing muscle anabolism and strength—including protein supplementation, testosterone supplementation, or a progressive low‐intensity in‐home rehabilitation training programme (3 days per week for 4 weeks)—can slightly improve functional recovery and may reduce 30‐day readmission rates. However, no significant differences were observed in weight, fat mass, muscle mass, or appendicular lean mass changes across groups or between all active intervention groups and the placebo group in geriatric patients following acute hospitalization for a medical illness [16]. This background sets the stage for the current experiment, addressing the gap identified and proposing new avenues for exploration.

We examined the effect of a multicomponent exercise programme on physical function, muscle mass and muscle quality assessed by ultrasound among vulnerable older patients admitted to an acute care geriatrics unit. We hypothesized that participation in a brief tailored exercise regimen (i.e., 3–5 days) would attenuate muscle function and structure changes compared with usual hospital care alone.

## 2 Methods

The trial (Prevention of Functional and Cognitive Impairment Through a Multicomponent Exercise Program) was a multicentre randomized clinical trial (RCT) conducted in Spain. Details on rationale and design were published previously [14]. In brief, older patients admitted to the acute care for the elderly unit were assessed for eligibility (Hospital Universitario de Navarra of Pamplona, Hospital Central de la Cruz Roja of Madrid and Complejo Hospitalario Universitario of Albacete). Inclusion criteria were age 75 years or older, a Barthel Index score of at least 60 points (range, 0 [total dependence] to 100 [total independence]) before admission, ability to ambulate with or without assistance and ability to communicate and cooperate with research staff. Exclusion criteria included expected hospital stay of less than 6 days, very severe cognitive decline (i.e., Global Deterioration Scale score = 7), terminal illness, uncontrolled arrhythmias, acute pulmonary embolism, recent myocardial infarction, recent major surgery, or extremity bone fracture in the past 3 months. Participants were randomized 1:1 to receive a multicomponent exercise programme or hospital usual care using computer‐generated assignments. Endpoint assessments were performed by trained individuals who were blinded to group allocation. The local ethics committee approved the protocol (Pyto2018/7) and written informed consent was obtained from participants or legal surrogates. This report followed Consolidated Standards of Reporting Trials (CONSORT) guidelines (Data S1).

## 3 Interventions

Acutely hospitalized older patients meeting the inclusion criteria were randomly assigned to either the intervention or control (usual care) group within the first 48 h of admission.

### 3.1 Usual Care

The control group received standard hospital care, including physical rehabilitation focused primarily on gait training if needed as decided by the attending medical team.

### 3.2 Multicomponent Exercise Programme

The individualized structured exercise programme was administered for three to five consecutive days, consisting of two daily 20‐min sessions (morning and evening) and based on the Vivifrail multicomponent physical exercise programme to prevent frailty, weakness and falls in older adults [17]. The morning sessions involved supervised progressive resistance training, balance and walking exercises tailored to the individual's functional status using variable resistance training machines (Matrix; Johnson Health Tech and Exercycle S.L., BH group). Upper (i.e., bench press) and lower body strengthening exercises (i.e., squads, leg press and knee extension) were performed at a load equivalent to 30% to 60% of the one‐repetition maximum (1RM) calculated at admission, with 2 to 3 sets of 8 to 10 repetitions. Participants were instructed to perform exercises at high speed for optimized muscle power output, with proper execution ensured. In the evening, participants practiced unsupervised exercises using elastic bands and mobility exercises adapted from physical exercise guide ‘Vivifrail’ [17].

Patients initiated exercise upon approval of clinicians when hemodynamically stable and judged able to participate.

## 4 Outcomes

The co‐primary endpoints were change in physical function from hospital admission to discharge assessed by the short physical performance battery (SPPB), 6‐m usual gait velocity test (GVT), dual‐task GVT and handgrip strength. Secondary endpoints were changes in ultrasonography measures associated with sarcopenia [18], including rectus femoris thickness, cross‐sectional area (CSA), echogenicity, subcutaneous adipose thickness (SAT) and intramuscular adipose tissue (IMAT).

### 4.1 Physical Function Assessments

The SPPB (range 0–12, lower worse) assessed usual gait speed over 4 m, standing balance testing and 5 repeated chair rises [19]. For the 6‐m GVT, participants walked at their self‐selected usual pace. Dual‐task GVT also measured gait velocity while participants counted backward from 100 or named animals aloud (verbal fluency). Isometric handgrip strength was measured in the dominant hand with a digital grip strength dynamometer (TKK 5401 GRIP D; Takei Scientiﬁc Instruments Co., Ltd, Tokyo, Japan) in this parallel‐group randomized trial that measures between 5 and 100 kg of force. Patients were placed in a sitting position in a chair, with an elbow complete extension, and were asked to squeeze the handle as forcefully as possible for 3 s. After this, two valid trials followed, and the highest value was used as the data point.

### 4.2 Ultrasonography Muscle Measurements

**FIGURE 1:**
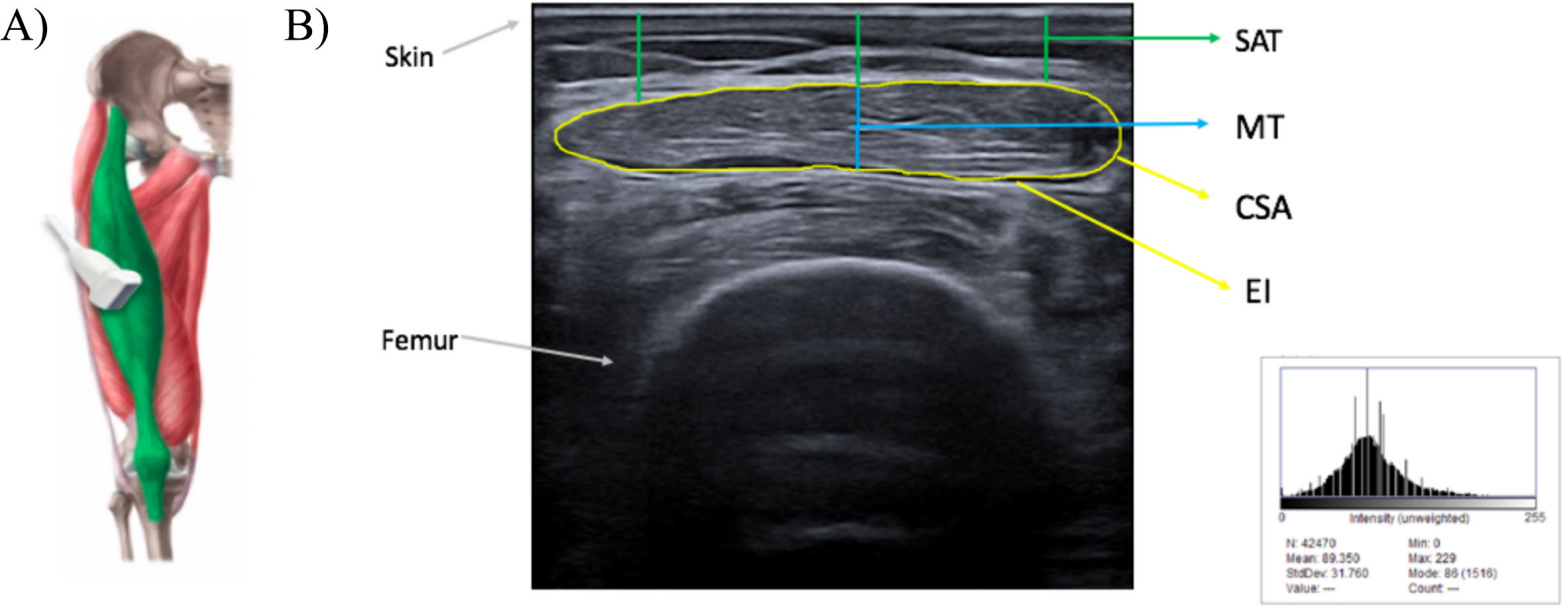
Illustrative representation of ultrasound parameters of the rectus femoris. (A) US image with representation of region of interest of the rectus femoris muscle. (B) Muscle ultrasound parameters (i) echo‐intensity and cross‐sectional area (yellow), (ii) muscular thickness (blue), and (iii) subcutaneous adipose thickness (green).

With participants lying supine with knees extended, rectus femoris architecture and echogenicity were assessed using B‐mode ultrasonography (Esaote MyLab 50; Esaote) with a 4‐ to 15‐MHz linear transducer by a trained operator (Y.G.A.) blinded to group assignment. Rectus femoris was selected based on previous evidence which highlighted the usefulness of this muscle measurement for sarcopenia screening even in older patients with functional and cognitive impairment [20]. Muscle assessments used fixed machine settings customized to optimize image resolution and consistency. Transmission gel facilitated acoustic contact with minimal pressure. Echogenicity, an indicator of intramuscular adiposity and fibrosis, was calculated by grayscale analysis (range, 0–255 arbitrary units [au]) within a region of interest (Figure 1) representing the rectus femoris CSA using validated software (ImageJ). (ImageJ, National Institutes of Health, USA, version 1.45). The transducer was positioned on the superior aspect of the thigh's long axis, at two thirds of the distance from the anterior superior iliac spine to the superior patellar border. The rectus femoris's inner outline was manually traced to calculate CSA using a movable cursor on a frozen image, distinguished by its hyperechoic appearance. Thickness was measured as the smallest distance between the superficial and deep aponeuroses. SAT was quantified as the distance between the fascial planes [21]. Estimated IMAT was calculated using a validated formula incorporating echogenicity and subcutaneous fat measures [21]. Ultrasonography reliably predicts muscle morphology compared with magnetic resonance imaging [21, 22].

### 4.3 Study‐Related Adverse Events

Falls, exercise programme interruptions and hospital stay modifications due to intervention characteristics were recorded.

## 5 Statistical Analysis

Between‐group and within‐group differences in mean change for continuous outcomes were assessed with linear mixed models adjusted for baseline values. Models included fixed effects for group, time and group‐by‐time interaction with participants fitted as random effects. For gait outcomes with asymmetric distribution, differences were evaluated with Poisson generalized mixed models. Analyses followed the intention‐to‐treat principle. The sample size was based on mobility outcomes reported previously [14]. Because the current aim was exploratory, no formal power calculation was performed. All tests were two‐sided (α = 0.05).

## 6 Results

**FIGURE 2:**
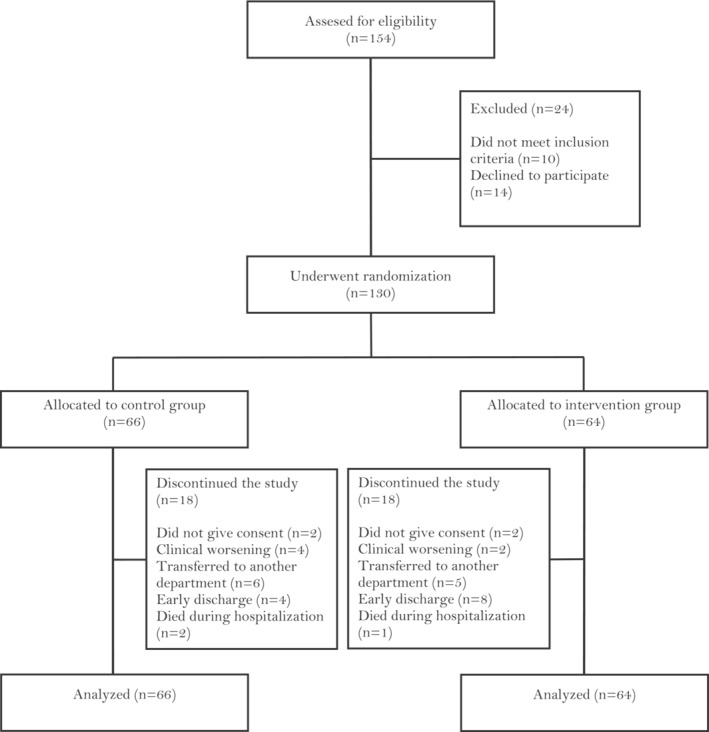
Study flow diagram.

Among 154 older patients screened, 130 were enrolled and randomized, including 57 (44%) women with a mean (SD) age of 87.7 (4.6) years (range, 75–101 years) (Figure 2). The groups did not differ significantly in any baseline characteristics or length of stay (median, 8 days for both; Table 1).

**TABLE 1:** Clinical and demographic characteristics of the participants.

Variable	Control group (*n* = 66)	Intervention group (*n* = 64)
Demographic data		
Age, years	88.4 (4.5)	87.1 (4.7)
Women, *N* (%)	30 (45.5)	27 (42.2)
Body mass index, kg/m^2^	26.3 (5.2)	26.8 (4.9)
Clinical data		
Barthel index, score	89.3 (10.9)	89.5 (11.6)
CIRS score, median (IQR)	13.0 (8.0)	13.0 (7.0)
Sarcopenia based on EWGSOP		
SPPB, score	4.3 (2.6)	4.7 (2.8)
Sarcopenia, *n* (%)	52 (78.8)	52 (81.3)
GVT, m/s	0.5 (0.2)	0.6 (0.3)
Sarcopenia, *n* (%)	48 (72.7)	46 (71.9)
Handgrip strength, kg	15.8 (6.1)	15.9 (6.7)
Sarcopenia, *n* (%)	51 (77.3)	51 (79.7)
Other endpoint measures		
Verbal GVT, m/s	0.5 (0.7)	0.5 (0.2)
Arithmetic GVT, m/s	0.4 (0.2)	0.4 (0.2)
Admission reason, *N* (%)		
Cardiovascular	17 (26)	18 (28)
Infectious	28 (42)	25 (39)
Pulmonary	3 (5)	4 (6)
Gastrointestinal	6 (9)	5 (8)
Neurological	5 (8)	5 (8)
Other	7 (10)	7 (11)

*Note:* Data are mean (SD) unless otherwise stated.

Abbreviations: CIRS, Cumulative Illness Rating Scale; EWGSOP, European Working Group on Sarcopenia; GVT, gait velocity test; IQR, interquartile range; SPPB, short physical performance battery.

### 6.1 Physical Function Outcomes

**FIGURE 3:**
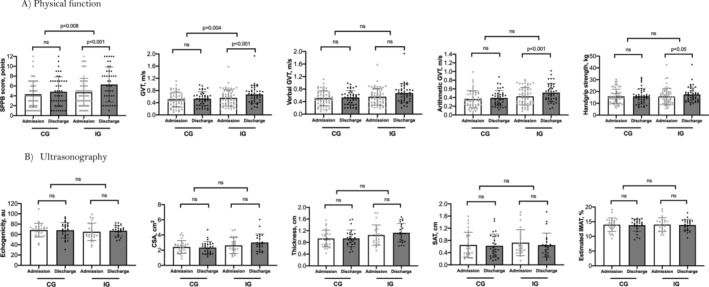
Individual physical function and ultrasonography measurement values at admission and discharge by study groups and between group comparisons.

From hospital admission to discharge, the exercise group increased their SPPB score by 0.98 points (95% CI, 0.28–1.69 points) more than the usual care controls (intragroup mean change, 1.30 vs 0.32 points; *p* = 0.008) (Table 2). Similarly, the exercise group improved their 6‐m usual gait velocity by 0.09 m/s (95% CI, 0.03–0.15 m/s) more than controls (intragroup mean change, 0.08 vs − 0.004 m/s; *p* = 0.004). Although dual‐task gait velocity was no different, the exercise group made fewer errors on verbal fluency while walking at discharge (mean difference, 0.30 words; 95% CI, 0.10–0.93; *p* = 0.037). Although the difference in handgrip strength between groups did not reach statistical significance, there was a notable improvement in the intervention group at discharge. This trend was not mirrored in the control group, suggesting a positive direction of effect (mean difference, 1.19 kg; 95% CI, 0.01–2.37; *p* = 0.052). Within group pre‐post physical function values are reported in Figure 3 and Table S1.

**TABLE 2:** Results of primary endpoints by group.

Endpoints	∆ Control group	∆ Exercise group	Between‐group difference (95% CI)	*p* between groups
SPPB, score	0.32 (−0.17, 0.81)	1.30 (0.80, 1.81)	0.98 (0.28, 1.69)	0.008
GVT, m/s	−0.004 (−0.05, 0.04)	0.08 (0.04, 0.13)	0.09 (0.03, 0.15)	0.004
Verbal GVT				
Velocity, m/s	−0.02 (−0.06, 0.02)	0.02 (−0.02, 0.06)	0.05 (−0.01, 0.10)	0.111
Correct answers, score^a^	1.06 (0.89, 1.26)	1.09 (0.91, 1.29)	1.03 (0.80, 1.31)	0.836
Errors, score^a^	0.91 (0.42, 1.95)	0.27 (0.12, 0.63)	0.30 (0.10, 0.93)	0.037
Arithmetic GVT				
Velocity, m/s	0.02 (−0.02, 0.05)	0.06 (0.03, 0.09)	0.04 (−0.004, 0.09)	0.078
Correct answers, score^a^	1.05 (0.92, 1.20)	0.97 (0.83, 1.13)	0.93 (0.75, 1.13)	0.425
Errors, score^a^	1.01 (0.78, 1.30)	1.17 (0.87, 1.59)	1.16 (0.78, 1.72)	0.447
Handgrip strength, kg	−0.24 (−1.08, 0.60)	0.94 (0.12, 1.78)	1.19 (0.01, 2.37)	0.052

*Note:* Data in each group are expressed as change from baseline (admission) to discharge (mean and 95% confidence interval).

Abbreviations: GVT, gait velocity test; SPPB, short physical performance battery.

^a^ Poisson mixed model.

### 6.2 Muscle Structure Outcomes

No significant differences were noted between the exercise and control groups for changes in any ultrasound measure of rectus femoris characteristics, including echogenicity (mean difference, 2.26 arbitrary units [au]; 95% CI, −5.34 to 0.87 au; *p* = 0.56), CSA (mean difference, 0.46 cm^2^; 95% CI, −0.12 to 1.04; *p* = 0.12), thickness (mean difference, 0.07 cm; 95% CI, −0.09 to 0.24; *p* = 0.37), SAT (mean difference, −0.05 cm; 95% CI, −0.22 to 0.12; *p* = 0.58), or estimated IMAT (mean difference, 0.04%; 95% CI, −1.14% to 1.21%; *p* = 0.95) (Table 3). Within group pre‐post ultrasonography measurement values are reported in Figure 3 and Table S1. At discharge, 56.8% participants in the intervention group were sarcopenic based on the SPPB, 75.6% for the GVT and 81.8% for handgrip strength, whereas higher prevalence was observed in the control group (82.6% for SPPB, 83.3% for GVT and 83.7% for handgrip strength) according to EWGSOP criteria. The findings of the study are summarized in Figure S1. No study‐related adverse events occurred, and no one had to discontinue exercise for safety concerns.

**TABLE 3:** Results of secondary endpoints by group.

Endpoints	∆ Control group	∆ Exercise group	Between‐group difference (95% CI)	*p* between groups
Echogenicity, au	−0.17 (−5.33, 4.99)	2.09 (−3.50, 7.68)	2.26 (−5.34, 0.87)	0.563
CSA, cm^2^	−0.08 (−0.48, 0.31)	0.38 (−0.05, 0.80)	0.46 (−0.12, 1.04)	0.125
Thickness, cm	0.01 (−0.10, 0.11)	0.08 (−0.04, 0.20)	0.07 (−0.09, 0.24)	0.372
SAT, cm	−0.03 (−0.15, 0.09)	−0.08 (−0.21, 0.05)	−0.05 (−0.22, 0.12)	0.579
Estimated IMAT, %	−0.17 (−0.97, 0.63)	−0.14 (−1.00, 0.73)	0.04 (−1.14, 1.21)	0.951

*Note:* Data in each group are expressed as change from baseline (admission) to discharge (mean and 95% confidence interval). Ultrasonography data correspond to 27 participants in the control group and 23 in the exercise group.

Abbreviations: au, arbitrary units; CSA, cross sectional area; IMAT, intramuscular adipose tissue; SAT, subcutaneous adipose thickness.

## 7 Discussion

In this parallel‐group randomized trial of acutely ill hospitalized older adults, a brief 3‐ to 5‐day tailored exercise programme emphasizing progressive muscle power training significantly improved physical function per the SPPB and usual gait velocity compared with hospital usual care. However, exercise did not alter ultrasonography measures of quadriceps muscle or fat morphology. The results provide further evidence that appropriately designed exercise should be a vital component of inpatient care to prevent disability.

Our findings indicate that even very brief physical exercise sustained over just 3 to 5 days, when tailored appropriately to individual ability, can elicit meaningful improvements in physical function among medically complex older inpatients compared with usual care. Specifically, the structured Vivifrail exercise programme yielded significant benefits in overall physical performance on the SPPB and usual gait velocity. Our results align with much data showing that structured exercise training is essential to prevent hospital‐acquired disability, whereas basic mobility interventions may be inadequate [12, 13, 14]. Meta‐analyses define optimal parameters to maximize functional responses from acute exercise [23]. Just 1 session of resistance training can attenuate disuse muscle atrophy during bed rest in older adults [24]. Moreover, A single bout of progressive resistance training can increase the muscle protein anabolic response in older adults [25, 26], and muscle protein synthesis has been shown to correlate with increased skeletal muscle thickness [24] and myofiber CSA [27]. Exercise, particularly progressive strength training, appears to mitigate disuse‐induced muscle atrophy and may delay the onset or progression of sarcopenia, potentially reducing healthcare costs and enhancing the quality of life for the older population [24]. Longitudinal studies have documented significant muscle mass losses during hospitalization in acutely hospitalized patients, which correlate with increased in‐hospital mortality, extended hospital stays and higher rates of readmission [9]. In our investigation, no participants in the control group exhibited muscle atrophy or functional decline throughout the hospital stay. The scope of our study focused on the effects of short‐term hospital stays (3–5 days), during which participants received as‐needed physical rehabilitation. Moreover, the ACE unit's geriatricians were particularly vigilant about the risks associated with prolonged bed rest and endeavoured to prevent hospital‐associated disabilities to the greatest extent feasible.

Although we observed functional improvements with training, ultrasonography detected no favourable between‐group differences in rectus femoris morphology, including mass, architecture, echogenicity, or fat infiltration. During a two‐week hospitalization, rectus femoris CSA was reduced by an average of 13.6%. Muscle CSA changes, as assessed by ultrasonography during hospital stays, can be instrumental for prognosis risk assessment in patients with acute heart failure [28]. While bed rest studies have documented measurable muscle function changes within days [4], the specific exercise dosing and timing required to significantly impact muscle structure may vary among hospitalized patients. For example, in‐hospital resistance training has been shown to favourably affect muscle function in patients with chronic obstructive pulmonary disease by influencing the anabolic‐catabolic balance and decreasing myostatin levels [29]. Furthermore, intensive strength training has been observed to promptly improved muscle quality in older adults living in the community [30]. While our findings did not demonstrate significant enhancements in muscle ultrasonography measurements, they do suggest that an exercise regimen might avert the decline typically associated with acute hospital stays. This implication is particularly noteworthy, considering literature that highlights muscle mass and function deteriorations even during short‐term immobility. Our findings lend support to the effectiveness of the exercise programme in maintaining muscle health during hospitalization.

In addition to mobility gains, acute exercise (i.e., 3–5 days) yielded cognitive benefits for verbal fluency during dual‐task walking. Hospitalization commonly impairs cognition and physical function in tandem via shared pathways [31]. In‐hospital exercises seem essential during hospitalization, providing significant cognitive gains beyond usual care [32]. Previously, it was shown that a similar multicomponent exercise intervention composed of 5 to 6 exercise days improved cognition (i.e., executive function and verbal fluency domains) in older patients hospitalized with acute medical conditions [33]. How exercise‐induced neurological and muscular adaptations interact to improve cognition warrants investigation. Studying these mechanisms may refine interventions to maximize outcomes.

Our study has several limitations. Firstly, this exploratory analysis was not the primary focus of the original multicentre randomized trial; thus, the sample size was calculated based on different predefined endpoints [14]. Secondly, many limitations related with the ultrasound technique used in this study should be mentioned. The equation used to explore IMAT content was validated with participants with lower body mass index (mean 23 kg/m^2^) compared with our study population (mean 26 kg/m^2^). Moreover, there is an important variability inherent to the measure itself. In order to reduce this variability, all assessments were performed by a trained operator using a standardized protocol. Additionally, previous research has highlighted that echogenicity may not be an accurate measurement of IMAT content [34]. Results may not generalize to older adults with poorer functional status at baseline, as we only included patients able to walk independently with a Barthel Index score of at least 60 points before hospitalization. Those unable to ambulate were excluded. Finally, we only examined short‐term changes over a brief 3‐ to 5‐day exercise programme. Findings may not extend to patients with longer hospital stays.

However, strengths include the novelty, as this research represents the first study to our knowledge investigating effects of acute in‐hospital exercise (i.e., 3–5 days) on muscle morphology measured by ultrasonography in older patients. The mobility improvements observed in the absence of detectable muscle changes suggest that other neurological or physiological adaptations likely mediate functional gains. Previous evidence has demonstrated that neural adaptations represent the first positive adaptations in response to resistance training [35, 36, 37]. In fact, the increases in muscle function and muscle power with progressive resistance training occur before and exceed the hypertrofic morphological response, and this is explained by the early physiological phase of neuroadaptation that frequently follows the first days of training [37, 38] which could translate into better functional capacity. Finally, this trial also enrolled a relevant but often excluded population of older adults with multiple chronic conditions, cognitive impairment and even dementia.

## 8 Conclusions

Among vulnerable older patients hospitalized with acute illness, participation in a brief tailored multicomponent exercise programme elicited improvements in physical function and mobility. Contrary to our hypotheses, there was no evidence that a short, individualized exercise program (i.e., 3–5 days) led to changes in muscle structure compared to habitual hospital care. The functionally meaningful benefits of appropriately tailored exercise interventions underscore their vital role during hospitalization for attenuating or preventing acute sarcopenia and associated disability. Further trials must define time‐dependent effects on long‐term outcomes.

## Ethics Statement

All patients or their legal representatives provided written consent. Ethics approval was granted by the Hospital Universitario de Navarra Research Ethics Committee (Pyto2018/7) [39]. The study followed the principles of the Declaration of Helsinki, and all the authors certify that they comply with the ethical guidelines for publishing in the *Journal of Cachexia, Sarcopenia and Muscle: update 2019*.

## Conflicts of Interest

The authors declare no conflicts of interest.

## Supporting information


**Data S1.** Supporting Information
